# Coupling Liquid Chromatography to Orbitrap Isotope Ratio Mass Spectrometry: Overcoming Isotope Effects of Chromatography and Amount-Dependency by Peak Homogenization

**DOI:** 10.1021/acs.analchem.5c05530

**Published:** 2025-12-19

**Authors:** Aoife Canavan, Leonhard Prechtl, Habib Al-Ghoul, Nils Kuhlbusch, Andrea M. Erhardt, Martin Elsner

**Affiliations:** † TUM School of Natural Sciences, Department of Chemistry, Chair of Analytical Chemistry and Water Chemistry, Technical University of Munich, Lichtenbergstraße 4, Garching 85748, Germany; ‡ 443714 Thermo Fisher Scientific (Bremen) GmbH, Hanna-Kunath-Straße 11, Bremen 28199, Germany; § Department of Inorganic and Analytical Chemistry, University of Münster, Corrensstraße 48, Münster 48149, Germany; ∥ Department of Earth and Environmental Sciences, University of Kentucky, 121 Washington Avenue, Lexington, Kentucky 40506, United States

## Abstract

Electrospray ionization Orbitrap mass spectrometry (ESI-Orbitrap-MS) has recently proven to be a powerful tool for compound- and position-specific stable isotope analysis, targeting isotopologues of multiple elements (H, C, N, O, S) in polar analytes. However, studies have so far mainly focused on pure analyte solutions via direct infusion. Here, we present the online coupling of liquid chromatography (LC) to ESI-Orbitrap-MS for stable isotope analysis of sulfamethoxazole (SMX), a synthetic antimicrobial, used as a model compound. Our study explored the fidelity of isotope values with two strategies for capturing and broadening chromatographic peaks after LC. When capturing the target analyte in a capillary (0.7 mm inner diameter), peak shape was retained, resulting in systematic deviations in isotope values caused by isotope effects from chromatography of up to 70‰ and amount-dependency of up to 65‰. A dynamic mixing chamber was used to homogenize the target analyte upon elution, removing these deviations and resulting in stable carbon, nitrogen, and sulfur isotopologue ratios in various fragments of sulfamethoxazole across the chromatographic peak. Comparison with data from conventional magnetic sector isotope ratio mass spectrometry successfully calibrated the setup for carbon and sulfur isotope ratio analysis. This resulted in a precision for δ^13^C within the isoxazole moiety from SMX of 1.5‰ (95% confidence intervals CIs derived from uncertainties of sample (*n* = 4) and reference (*n* = 5)), and for δ^34^S in the SO_2_ fragment of 0.9‰ (95% CI, derived again from uncertainties of sample (*n* = 4) and reference (*n* = 5)). This work paves the way for the online coupling of LC to ESI-Orbitrap-MS in future compound- and position-specific stable isotope analyses across various research fields.

## Graphical abstract

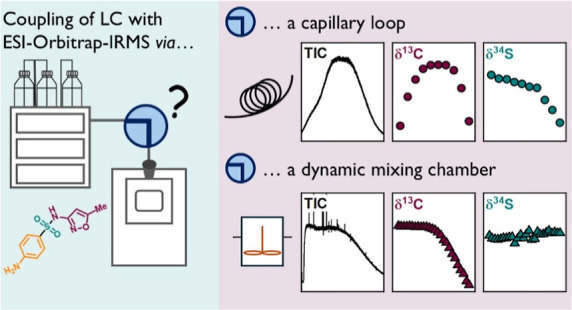

## Introduction

Stable isotope analysis of light elements (C, H, O, N, S) is a powerful tool for uncovering the origin, transformation, and fate of organic compounds by revealing detailed insights into their sources, (bio)­chemical formation pathways, and degradation mechanisms.^1–4^ This methodology has broad applications across diverse scientific disciplines, including environmental chemistry,^4–10^ food authentication,^11–14^ and forensic science.^15–17^ Isotope ratio measurements of complex organic molecules typically require converting the analyte into a simple gas molecule, such as CO_2_ for ^13^C/^12^C, N_2_ for ^15^N/^14^N, and SO_2_ for ^34^S/^32^S analysis, before measurement with a sector field isotope ratio mass spectrometer (IRMS).^18^

For compound-specific isotope analysis (CSIA), gas or liquid chromatography is hyphenated with IRMS (GC-IRMS, LC-IRMS) to measure the isotopic signature of target analytes.^19,20^ However, GC-IRMS, the commonly used approach for carbon and nitrogen isotope analysis, is restricted to volatile and semivolatile compounds. LC-IRMS, on the other hand, is primarily limited to carbon isotope analysis and—in the absence of tailor-made solutions—is incompatible with widely used carbon-containing eluents.^21,22^ Beyond instrumental challenges, a fundamental disadvantage of CSIA by GC- and LC-IRMS is that analyte conversion to the respective measurement gas only yields the average isotopic composition of a molecule. This process results in the rescrambling of multiply substituted isotopologues, which hinders clumped isotope analysis. Furthermore, this can dilute meaningful isotope effects occurring at a specific position within the target analyte, which is particularly pronounced in large molecules. Such position-specific isotope information could provide valuable insights during degradation processes when the isotopic signature at the reactive site changes, whereas isotope ratios at other, nonreactive positions still represent the compound’s origin.^23^

Existing techniques for position-specific isotope analysis are (i) nuclear magnetic resonance spectroscopy, (ii) GC-pyrolysis-GC-IRMS, and (iii) high-resolution, multicollector gas source mass spectrometry (MCMS). However, these techniques show limitations. First, they rely on the introduction of compounds in pure form, thereby forfeiting the advantage of chromatographic separation to isolate substances from complex mixtures, which is a hallmark of CSIA. Second, they either require micromoles of sample for NMR or are restricted to specific small analytes, such as acetic acid (GC-pyrolysis-GC-IRMS) and short-chain alkanes (MCMS).^24–26^

Orbitrap mass analyzers, known for their high resolution and accuracy, have gained recent attention for their ability to simultaneously determine intramolecular isotope ratios of various elements.^18,27–29^ The minimum sample size of the target analyte is smaller than that of NMR (nanomoles instead of micromoles). Then, electrospray ionization (ESI) enables the introduction of aqueous solutions, broadening the range of analytes compared to GC-IRMS. Further, analyte molecules are introduced as intact ions, preserving their molecular structure, unlike in GC- and LC-IRMS, where conversion to CO_2_, N_2_, etc. is required. Consequently, analytes can be fragmented—either directly after ionization or after preselection of ions in a quadrupole within a subsequent collision cell, where selected ions can be accelerated and brought into collision with neutral gas molecules. This process provides fragment-specific isotope information, which subsequently can be used to calculate the isotope ratios of specific molecular sites.

A growing number of studies have explored the development and assessment of the Orbitrap-MS for measuring isotope ratios in polar compounds. Orbitrap-based CSIA was conducted for fatty acids, methanesulfonate, and oxyanions, allowing for the simultaneous study of multiple elements.^30–36^ A recent study demonstrated precise measurements of carbon isotope values of perfluorooctanoic acid.^37^ For amino acids, both molecular average and position-specific carbon isotope analyses were performed.^27,38–42^ Orbitrap-MS CSIA of acetate allowed differentiation of metabolic pathways in bacteria and tapped for the first time the hydrogen isotope exchange within acetate to constrain its geological lifetime.^43,44^ A technique to measure methyl phosphonic acid indicated potential source substrates, synthetic routes, and possible degradation mechanisms.^45^ Finally, Orbitrap-derived carbon isotope values of polycyclic aromatic hydrocarbons from the asteroid Ryugu and the Murchison meteorite could indicate different formation environments.^46^

However, in the absence of chromatographic separation, a critical bottleneck to applications is still the isolation of the target compound. Compared to conventional analysis, this is even more important, because isotope artifacts in the Orbitrap-MS can arise from interferences during ionization or space-charge effects.^45,47^ While coupling with chromatography has been brought forward for GC-Orbitrap-MS, such a solution is still missing for liquid chromatography. In GC-Orbitrap-MS, peak broadening proved to be essential for obtaining isotope measurements with high precision, as accomplished through capturing the target analyte in a post-GC reservoir before isotope measurement.^39,48^ An analogous setup for liquid chromatography, including peak capturing and broadening, is, hence, of great interest to spearhead precise isotope measurements of polar, nonvolatile analytes by ESI-Orbitrap-MS.

Coupling LC with Orbitrap-MS can be challenged, however, by two kinds of potential artifacts. First, partitioning isotope effects are at work during chromatographic separation, leading to slightly different retention times.^21,49–51^ Such effects were not observed in GC-Orbitrap-MS, indicating effective mixing within the reservoir. Whether this is also the case in the liquid phase, where diffusivities are lower by 5 orders of magnitude, remains to be investigated. Second, linearity tests are a hallmark of isotope analysis: they ensure that isotope values are concentration-independent and can, thus, be accurately integrated over different concentration regimes of a chromatographic peak. However, analyte–analyte interactions during ionization by ESI and inside the mass analyzer may potentially lead to nonlinearity, as demonstrated for the oxyanion nitrate: the linear range for ^15^N/^14^N extended down to 25 μM, below which measurements had to be accomplished by matching concentrations of sample and reference.^31^

It was, hence, the aim of our study to explore whether isotope values showed artifacts from chromatography; whether they were, in addition, concentration-dependent across a chromatographic peak; and whether such interferences could be avoided by peak capturing and homogenization, similar to the peak broadening reservoir in GC-Orbitrap-MS. We chose the synthetic antibiotic sulfamethoxazole (SMX) as a model compound due to its widespread use and potential ecological impact, as demonstrated by numerous CSIA studies investigating its various degradation pathways.^52–57^ The objectives of this work were (i) to investigate the linear range at concentrations between 1 and 10 μM for different fragments of SMX; (ii) to assess hyphenation of Orbitrap-MS with LC by capturing SMX within a capillary loop (0.7 mm inner diameter); (iii) to investigate whether isotope effects of chromatography would be observable in the captured peak leading to artifacts; (iv) to explore whether a nonlinear behavior of isotope values at low concentrations would affect the isotope values after peak capture; (v) if so, to develop strategies to avoid such effects; and last (vi) to validate the accuracy of this setup by measuring ratios of ^13^C/^12^C and ^34^S/^32^S within fragments of SMX and comparing them with conventional magnetic sector IRMS techniques.

## Experimental Section

### Chemicals and Sample Preparation

Sulfamethoxazole (analytical standard, different batches) and methanol (as Orbitrap eluent: “hypergrade for LC–MS”; for HPLC cleanup: “HPLC-grade”) were purchased from Merck (Germany); formic acid (ROTIPURAN ≥99.0%, LC–MS grade) was from Carl Roth (Germany). The used water (18.2 MΩ cm at 25 °C) was from a Milli-Q Reference water purification system (Merck Millipore, USA). All standard solutions of SMX for ESI-Orbitrap measurements were prepared in a mixture of 36% MeOH in H_2_O with 0.1% formic acid and stored at 4 °C until further use. When solvents were used in HPLC pumps without degassing units, they were degassed by applying a vacuum during ultrasonication for 15 min. Standards for stable carbon isotope analysis were prepared by mixing a solution of SMX at natural abundance with ^13^C-labeled SMX, which included the label positioned at a specific site within the isoxazole moiety (Section S1).^58^ The resulting mixtures were characterized by GC-IRMS (Section S2). For sulfur isotope analysis, SMX from different batches of commercial product was used and characterized by EA-IRMS (Section S3).

### Orbitrap Instrumentation

A Vanquish liquid chromatography system was coupled with an electrospray ionization Orbitrap Exploris 240 mass spectrometer (both, Thermo Fisher Scientific, Germany). The Vanquish liquid chromatography system consisted of a Split Sampler HT VH-A10-A, Binary Pump N VN-P10-A (“low-flow pump”), Binary Pump H VH-P10-A (“high-flow pump”), Diode Array Detector (DAD) FG VF-D11-A, and Column Compartment VH-C10-A (all, Thermo Fisher Scientific, Germany). The sample was introduced into the ESI source in two ways: either by the low-flow pump via the autosampler of the Vanquish system (total loop volume: 130 μL, Thermo Fisher Scientific, Germany) or through a syringe pump (SKE 10, Chemyx Inc., USA) at a flow rate of 4 μL min^–1^. For ionization, the atmospheric pressure ion source OptaMax NG was used with a nonheated ESI probe equipped with a low-flow needle insert (Thermo Fisher Scientific, Germany).

The ionization mode, either positive or negative, was chosen depending on the target fragments. The ESI spray was optimized before each sequence to ensure a stable and intense TIC (relative standard deviation <8%) by adjusting the spray voltage, sheath gas (0–30), and auxiliary gas values (0–1). After ionization, the continuous beam of ions was filtered by an advanced quadrupole technology (AQT) mass filter, which allowed only ions within the set isolation window to pass. They were then transmitted through the curved linear trap (C-trap) and stored as packages in the ion-routing multipole (IRM), with their quantity being regulated by the automatic gain control (AGC) algorithm. The accumulated ions were transferred back through the C-trap into the Orbitrap mass analyzer for measurement. The oscillation of each ion package inside the analyzer was recorded as image current and stored as transient. By increasing the microscan setting, 10 transients were averaged prior to performing an enhanced Fourier Transform (eFT) to generate the mass spectrum. Fragmentation was induced by either collision-induced dissociation (CID) before ions entered the AQT mass filter (so-called source fragmentation), or, after passage of the AQT, by Higher Energy Collisional Dissociation (HCD) in the IRM. Accessible fragments for position- and fragment-specific isotope analysis were identified by increasing the energy for either CID or HCD while directly infusing an SMX solution (5 μM, 36% MeOH in H_2_O with 0.1% formic acid) with the syringe pump into the Orbitrap-MS (Figure S1). Fragments of interest included one representing the isoxazole moiety (F99), one representing the aromatic aniline part (F92), and one representing the SO_2_ group (F64) (Scheme 1). The optimal settings for each of these fragments were selected by choosing conditions that yielded the highest abundance of the fragment of interest, along with a resolution that would resolve the isotopologues of interest (Table 1 and Figure S2).

**Scheme 1:**
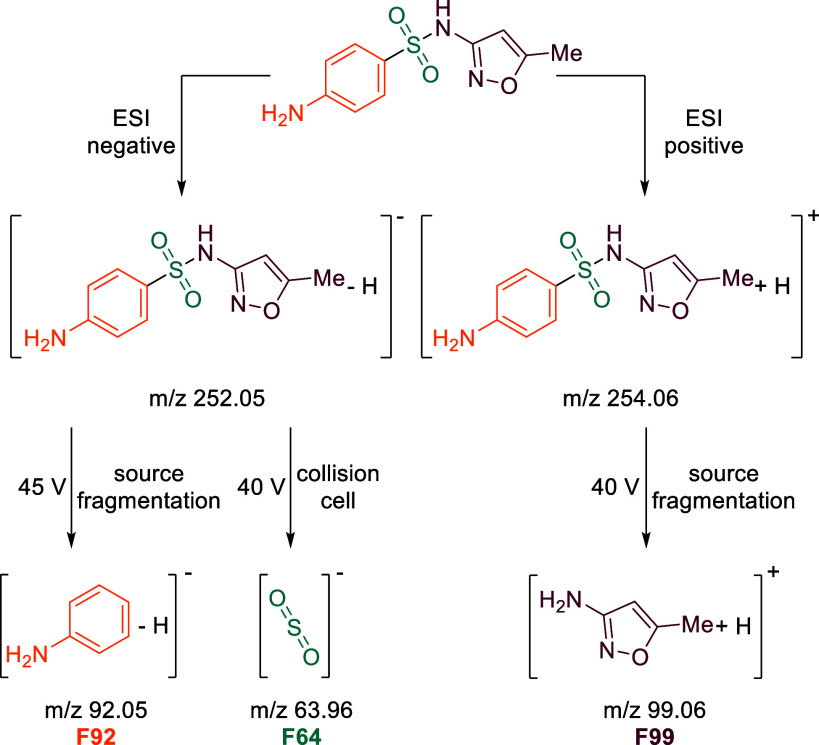
Target Fragments of Sulfamethoxazole after Positive or Negative Electrospray Ionization Using Source Fragmentation or HCD Fragmentation in the IRM

**Table 1:** Instrument Settings Used for Each Fragment of SMX

	F64	F92	F99
polarity (±)	negative	negative	positive
ion transfer tube temperature (°C)	320	320	320
nominal Orbitrap resolution	30,000	90,000	90,000
AQT isolation window (*m*/*z*)	249.5–254.5	88.5–95.5	94–104
absolute AGC target (#)	1.5 × 10^5^	1.5 × 10^5^	1.5 × 10^5^
microscans (#)	10	10	10
RF lens (%)	100	100	100
maximum ion injection time (ms)	1000	1000	1000
source fragmentation (yes/no)	no	yes	yes
source fragmentation energy (V)	-	45	40
HCD collison (yes/no)	yes^a^	no	no
absolute HCD collision energy (V)	40	-	-

a *m*/*z* 252 was selected as a precursor ion.

#### Determination of the Linear Range

For experiments investigating the amount-dependency of isotope analysis (“linearity experiments”), the autosampler was directly connected to the Orbitrap-MS without a separation column, using the low-flow pump under isocratic conditions (36% MeOH in H_2_O with 0.1% formic acid). For each injection, 80 μL of the respective SMX solution was injected at 4 μL min^–1^, resulting in ∼20 min of useable data for isotopologue ratio analysis. To ensure the absence of carryover, the flow rate was increased after 24 min to 20 μL min^–1^ and switched back to 4 μL min^–1^ after 29.9 min. The amount-dependency was tested by injecting solutions with varying concentrations (0.5 μM, 1 μM, 2 μM, 4 μM, 6 μM, 8 μM, and 10 μM) bracketed with injections of a 4 μM solution, which was used as a reference. Each concentration was measured in quintuplicate. To assess reproducibility, each linearity experiment was performed in duplicate for the fragment F64 and in triplicate for the fragments F92 and F99.

#### Comparison of Infusion Methods

To compare different injection techniques, alternating injections of the same SMX solution (5 μM, 36% MeOH in H_2_O with 0.1% formic acid) were applied either via the autosampler using the low-flow pump under isocratic conditions (36% MeOH in H_2_O with 0.1% formic acid) or via a syringe using the syringe pump at a flow rate of 4 μL min^–1^. Injections were alternated 3 times between the two injection methods for the sample application.

#### Automated Online Coupling of HPLC with ESI-Orbitrap-MS

To establish the coupling of HPLC with ESI-Orbitrap-MS, 20 or 40 μL of an SMX solution (100 μM) were injected. The chromatographic separation of SMX was performed on a C18 column (250 × 4.6 mm, Luna 5 μm C18(2) 100 Å, Phenomenex) with a flow rate of 0.5 mL min^–1^ under isocratic conditions (36% MeOH in H_2_O with 0.1% formic acid) using the high-flow pump. For subsequent isotope analysis using an ESI-Orbitrap-MS, the chromatographic peak was captured either in a stainless-steel capillary (0.7 mm inner diameter, 540 μL) or a dynamic mixing chamber (V7119-1, volume 1140 μL, Knauer, Germany) connected to an automatic 6-port, 2-position switching valve integrated into the column compartment (Valve 1 in Figure 1). The volume of the stainless-steel capillary was chosen to capture the entire peak of the target analyte (0.7 mm inner diameter, 540 μL). The operating principle of the dynamic mixing chamber relied on a Teflon-coated magnetic stirring bar, which was activated for 1 min upon capturing. The peak capture was automated for the dynamic mixing chamber by using a trigger based on the increase in the DAD signal at the retention time of SMX, which switched Valve 1 positions for 80 s and directed the target peak into the dynamic mixing chamber (Section S5). After capturing and homogenizing the peak, it was continuously delivered into the Orbitrap-MS by a low-flow pump at a flow rate of 4 μL min^–1^ under isocratic conditions (36% MeOH in H_2_O with 0.1% formic acid). The remaining LC effluent from the first dimension (high-flow pump) was diverted to waste rather than being routed to the capillary or the mixing chamber and the Orbitrap-MS. A second valve (Rheodyne MXP Injection Valve, Rheodyne, IDEX Health & Science, USA, Valve 2 in Figure 1) was used to bracket a reference from a syringe pump. Prior to the automation of this setup, an effective method for capturing SMX in the mixing chamber or capillary loop required the installation of a DAD detector after peak capture to optimize valve switching time for complete capture of the chromatographic peak, as used for the measurements shown in this manuscript. To determine the setup’s accuracy using the dynamic mixing chamber, each homogenized standard was bracketed with a 4 μM SMX solution from the syringe (36% MeOH in H_2_O with 0.1% formic acid) for drift correction. Valve 2 was switched every 20 min, resulting in 5 blocks of syringe measurements and 4 blocks of sample infusion.

**Figure 1:**
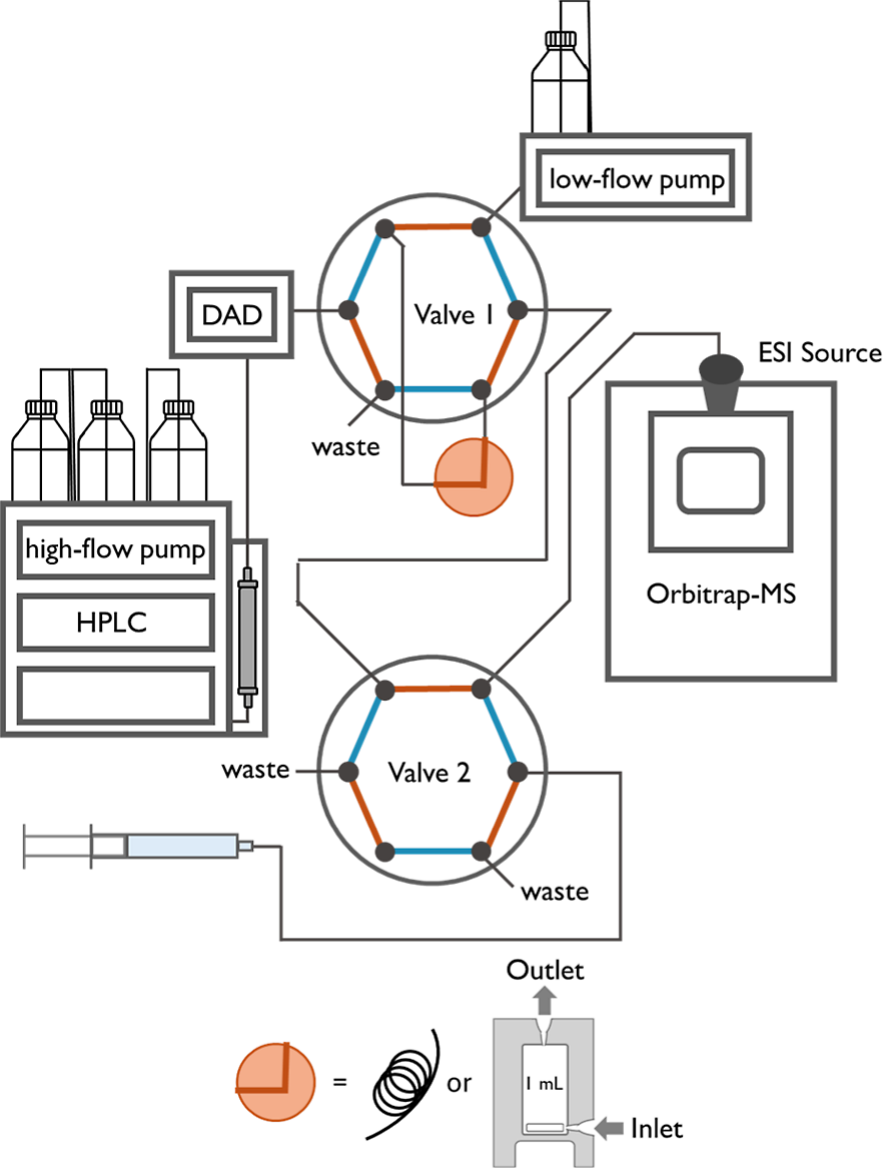
Schematic overview of the coupled HPLC-ESI-Orbitrap-MS system for stable isotope analysis.

### Data Processing

For all Orbitrap experiments, time intervals of 15 min were used for data processing. Ion intensities and noise for selected isotopic peaks were recovered from instrument.RAW files using IsoX (Thermo Fisher Scientific) or the IsoOrbi R package (version 1.3.9).^29^ The number of ions observed (IO) per scan *N*_IO_ was calculated by eq 1,^27^

1 $$N_{IO}=\frac{S}{N}\frac{C_{N}}{z}{\left(\frac{R_{N}}{R}\right)}^{1/2}\mu^{1/2}$$

Here, *S* is the ion intensity, *N* is the peak noise, *C*_N_ is an empirical constant measured by Makarov and Denisov^59^ relating the signal-to-noise ratio to ion counts (here: 3), *z* is the charge (here: 1), *R*_N_ is the reference resolution of 240,000, *R* is the nominal mass resolution, and μ is the number of microscans (here: 10). We extracted the peaks of the following ions: ^32^S^16^O_2_ (*m*/*z* 63.9623) and ^34^S^16^O_2_ (*m*/*z* 65.9581) for F64, ^12^C_6_^1^H_6_^14^N (*m*/*z* 92.0507), ^12^C_6_^1^H_6_^15^N (*m*/*z* 93.0477), and ^13^C_1_^12^C_5_^1^H_6_^14^N (*m*/*z* 93.0541) for F92, ^12^C_4_^1^H_7_^14^N_2_^16^O (*m*/*z* 99.0553), ^12^C_4_^1^H_7_^14^N^15^N^16^O (*m*/*z* 100.0524), and ^13^C_1_^12^C_3_^1^H_7_^14^N_2_^16^O (*m*/*z* 100.0587) for F99. The isotopologues containing only the most abundant isotopes are referred to as the basepeak of the respective fragments. The isotopologue ratios *R* ^*i*^ were calculated as the ratio of the summed number of ions observed *N*_IO_ of isotopologue *i* over the measurement time to those of the basepeak, according to Hilkert et al.^31^ or using the IsoOrbi R package (version 1.3.9)^29^ (eq 2).

2 $$R^{i}=\frac{\sum_{j=1}^{N}N_{IO}\left(isotoplogue\;i\right)}{\sum_{j=1}^{N}N_{IO}\left(basepeak\right)}$$

For comparison with IRMS, a stochastic distribution of multiply substituted isotopologues within the analyzed molecular or fragment ion was assumed. Under this assumption, the isotopologue ratios analyzed by Orbitrap-MS can be interpreted as the corresponding isotope ratio. (i.e., the ^13^C-substituted SMX ion over the unsubstituted SMX ion can be interpreted as ^13^C/^12^C isotope ratio). Isotope ratios are reported in the δ-notation in per mil (‰), relative to a reference using eq 3.

3 $$\delta^{h}E=\frac{R_{sample}-R_{reference}}{R_{reference}}$$

Here, δ^h^E is the δ-value of a given element, *R*_sample_ is the isotope ratio of the sample, and *R*_reference_ is the isotope ratio of the reference of the given element E. For isotope ratio measurements by Orbitrap-MS, a laboratory working standard of SMX was used as a reference. Further details on, for example, the respective concentration are provided for every single experiment in Section S8. Shifts of isotopic signatures are reported as the deviation Δδ^*h*^ *E*, between the isotope ratios in the sample (δ^*h*^ *E*_sample_) and a reference (δ^*h*^ *E*_reference_), according to eq 4.

4 $$\Delta \delta^{h}E={\delta^{h}E}_{sample}-{\delta^{h}E}_{reference}$$

Isotope values derived from EA- and GC-IRMS measurements are reported in the δ-notation in per mil (‰) relative to the international reference material Vienna PeeDee Belemnite (VPDB) for δ^13^C, air for δ^15^N, and Vienna-Canyon Diablo Troilite (VCDT) for δ^34^S by using eq 3.

## Results and Discussion

### Linearity Experiments

First, linearity experiments were conducted, where different concentrations were directly infused into the Orbitrap-MS using a low-flow pump connected to an autosampler, without the involvement of a chromatographic column. Each concentration was measured as a quintuplicate, and the calculated isotope values are reported as the deviations from the value of the 4 μM SMX solution (Figure 2). For Δδ^13^C in both fragments F92 and F99, a concentration-dependence was observed between 1 μM and 10 μM when referenced to a 4 μM SMX solution (Figure 2a,b). A similar nonlinear behavior at concentrations below 25 μM has been previously observed for δ^15^N in nitrate by Hilkert et al.^31^ In comparison, the Δδ^34^S_F64_ values in the linearity experiment showed a comparatively small concentration-dependency, with a linear range between 4 and 10 μM, and deviations of only up to 9‰ for concentrations below 4 μM (Figure 2c). Standard deviations were greatest for ^15^N isotope analysis of F92 and F99, compared to ^13^C and ^34^S, specifically for ^15^N in F99 (1‰–8‰) and F92 (3‰–27‰). The concentration-dependency of Δδ^15^N, in contrast, was smaller compared to Δδ^13^C in F92 and F99 (comparison of Figure 2a/d,b/e).

**Figure 2:**
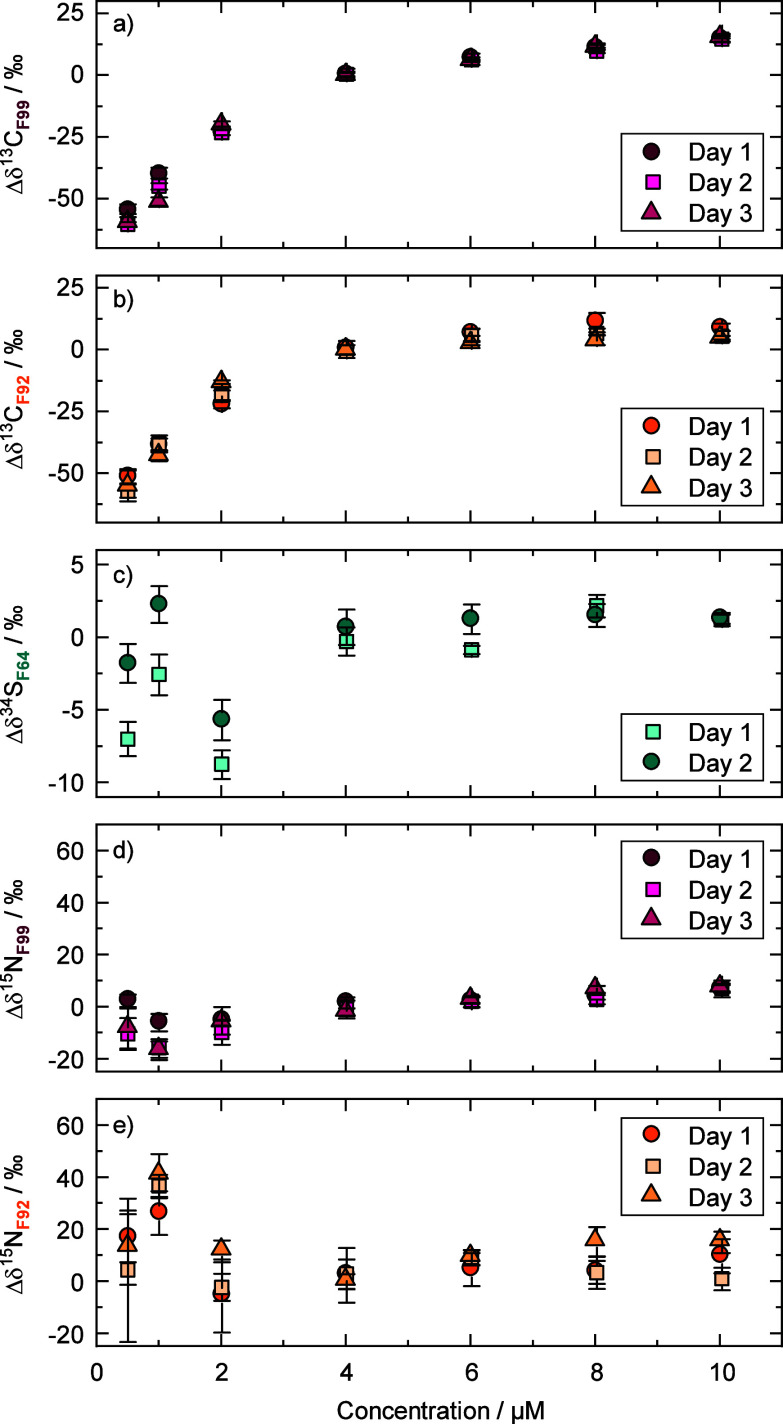
Linearity experiments, conducted by direct injection of SMX (i.e., bypassing the chromatographic column of Figure 1). Carbon, nitrogen, and sulfur isotope values of the respective fragments were obtained by infusing different concentrations in two or three different measurement series. These values are expressed as deviations from a 4 μM SMX solution, where each concentration was measured in quintuplicate and the respective error bars represent an estimate of the standard deviation of the measured quintuplicate.

### Using a Capillary for Analyte Capturing

We coupled an HPLC system with an Orbitrap mass analyzer via two 6-port valves (Figure 1). In this setup, the first valve was used to capture the peak of the target analyte after chromatographic elution in a capillary with an inner diameter of 0.7 mm. Subsequently, the target analyte was transferred to the Orbitrap-MS for isotope analysis at a flow rate of 4 μL min^–1^. The second 6-port valve enabled referencing by switching between SMX from a syringe pump (reference) and the HPLC (sample). Using this approach of peak capturing, we studied the isotope ratios of carbon and nitrogen in F92 and F99, as well as of sulfur in F64 (Figure 3). As shown in Figure 3a, the TIC of the measurements remained stable when SMX was injected via a syringe as a reference, whereas it took on the shape of a chromatographic peak when the SMX came from the captured fraction in the capillary loop. Both SMX were identical and, therefore, had the same isotopic composition. Hence, deviations from the average reference to the sample had to stem from artifacts. These deviations were calculated as Δδ^34^S, Δδ^13^C, and Δδ^15^N, respectively, relative to the syringe injection. Each 3 h measurement block was divided into segments of 15 min of data acquisition (Figure 3b–f).

**Figure 3:**
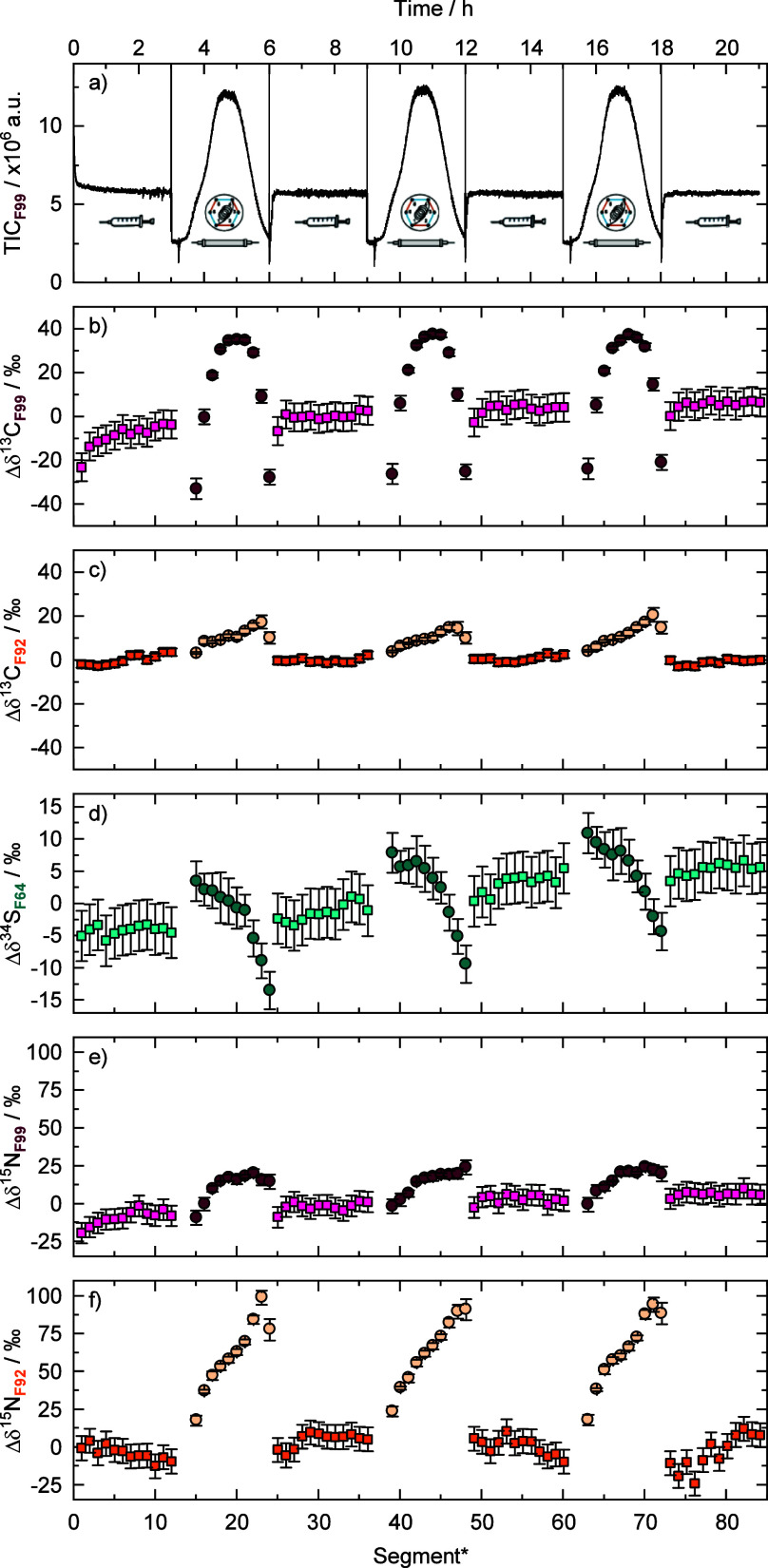
Peak capturing experiments using a capillary loop (0.7 mm) with the setup of Figure 1. (a) Representative TIC over time for F99 when alternating between reference from the syringe (4 μM) and sample from the captured peak inside the capillary (injection of 20 μL of a 100 μM SMX solution). (b–f) Deviations in the isotope values (Δδ^13^C, Δδ^34^S, and Δδ^15^N) across the respective chromatographic peaks compared to the average isotope value of the syringe injection as a reference for each fragment. The reported uncertainties were derived as described in the text. *each block was divided into segments of 15 min of data acquisition.

#### Observation of Non-Linearity

The Δδ^13^C_F99_ values of the captured SMX peak after LC in F99 were both more negative at the beginning and at the end of the captured peak, and more positive in the peak center compared to the reference (Figure 3b). Comparison with Figure 2a,b strongly suggests that this trend arose from a concentration-dependent (nonlinear) behavior within the chromatographic peak. Additionally, the Δδ^13^C_F92_ values in F92 showed a strong concentration-dependence in the independent linearity experiment (Figure 2b); however, in contrast to F99, the trend was not pronounced in Δδ^13^C_F92_ of the captured peak (Figure 2c). We note that a more thorough exploration of this phenomenon, for example, of effects caused by other interfering ions in the analyzed mass range, is beyond the scope of this study.

#### Observation of Chromatographic Effects on Isotope Values

The Δδ^34^S_F64_ in F64 from the captured SMX showed a consistent trend from negative to positive values over the chromatographic peak spanning a range of 20‰ (Figure 3d). Artifacts cannot arise solely from amount-dependency, as the linearity experiments only showed deviations up to 9‰. We hypothesize that this change in Δδ^34^S_F64_ values was primarily caused by isotope fractionation due to partitioning during liquid chromatography. A normal isotope effect was observed for ^34^S, meaning that the heavier isotope was retained more strongly than the lighter one (note that in our experimental setup, the end of the captured chromatographic peak was infused into the Orbitrap-MS first so that the peak order appears reversed in Figure 3). The existence of such chromatographic effects is well-established and has previously been observed in LC-IRMS, where isotope effects were inverse for ^13^C of caffeine, vanillin, and SMX.^21^ In the chromatography experiment for F92, a significant variation of ∼80‰ in the Δδ^15^N_F92_ of F92 was observed (Figure 3f), suggesting again isotope effects of chromatography. We hypothesize that this finding is likely associated with the compounds’ speciation. SMX has a p*K*_a_ value of 1.7, representing the protonation of its amine group in F92.^60^ The solvent mixtures used for samples and pumps contained 0.1% formic acid, resulting in a pH of ∼2.9. Hence, at a pH of ∼2.9, SMX exists in both protonated and neutral forms. Importantly, the existence of such partitioning isotope effects implies that it is not sufficient to measure only one part, but that the entire peak would have to be captured and analyzed to avoid losing information. This not only results in long measurement times but also emphasizes the problem of correcting simultaneously for amount-dependency across the peak, as highlighted for carbon in fragment F99.

#### Bracketing by Syringe Injection

We note that when both injection methods were compared—direct infusion with a syringe versus autosampler injection with the low-flow pump—no significant difference was observed for carbon isotope values in F99 and F92, sulfur isotope values in F64, and nitrogen isotope values in F99 (Figure S4a–d). However, for the nitrogen isotope values in F92, a deviation of up to 20‰ between the two different injection methods was found (Figure S4e), indicating that referencing by syringe is not suitable for absolute calibration, but rather to correct for instrument drifts over time such as reflected in the Δδ^34^S_F64_ values of the syringe injection in Figure 3d, and represented in the Allen plot of Figure S6.

#### Limitations of the Capillary for Peak Capturing

When connecting our HPLC system to the ESI-Orbitrap-MS via capture in a capillary and subsequent elution by a low-flow pump, it took 3 h to analyze the entire target analyte captured inside the capillary. On the other hand, when continuously infusing a reference SMX solution over 3 h by a syringe, the Allan deviation showed that the best improvement in precision was reached after 10 to 15 min, after which the loss by instrument drift was greater than the gain by counting statistics (Figure S6). When measuring over such time scales, frequent switching between reference and sample is, hence, essential to correct for instrumental drifts. However, such switching also results in sample loss and, thus, the loss of crucial information, particularly when isotope fractionation occurs during chromatography.

In addition, the isotope analysis of low concentrations at both the beginning and end of the peak is essential, since these parts of the peak contain valuable information related to isotope fractionation during chromatography. While this makes a correction for amount-dependency (“linearity”) mandatory, high day-to-day variations were observed in our linearity experiments that make such a daily correction extremely challenging. This, in turn, makes it impossible to correct for chromatographic effects by means of a weighted average across the peak. We therefore observed that capturing our SMX peak in a capillary (0.7 mm inner diameter) under the conditions used in this study did not result in a suitable workflow for isotope analysis. We recognize that further optimization of capillary diameters, flow rates, and critical MS parameters might improve the performance of this approach. Here, we instead explored a more universally applicable approach using a mixing chamber as detailed in the following section.

### Using a Mixing Chamber instead of the Capillary

To overcome the isotopic artifacts resulting from concentration-dependency (Figure 2) and chromatography (Figure 3), we bring forward online homogenization of the captured target analyte peak as a convenient solution. This was accomplished by replacing the capillary loop with a dynamic mixing chamber, which is normally used for stable mixing of organic eluents in binary LC gradients. The mixing chamber had a nominal volume of 1140 μL and comprised a magnetic stirring bar, which could be switched on and off. Upon capturing the SMX peak inside the chamber, it was homogenized by switching on the magnetic stirrer for 1 min. To investigate the effect, we subsequently eluted the homogenized peak into the Orbitrap-MS without standard bracketing. The TIC remained stable for ∼3 h and only afterward decayed due to dilution with the eluent (Figure 4a). Once this decline set in, this was also reflected in the nonlinear effects of isotope values (Figure 4b–f), which remained stable for the first 3 h before gradually decreasing. Again, this amount-dependency was not observed for the Δδ^34^S values, which is consistent with our results from Figure 2c.

**Figure 4:**
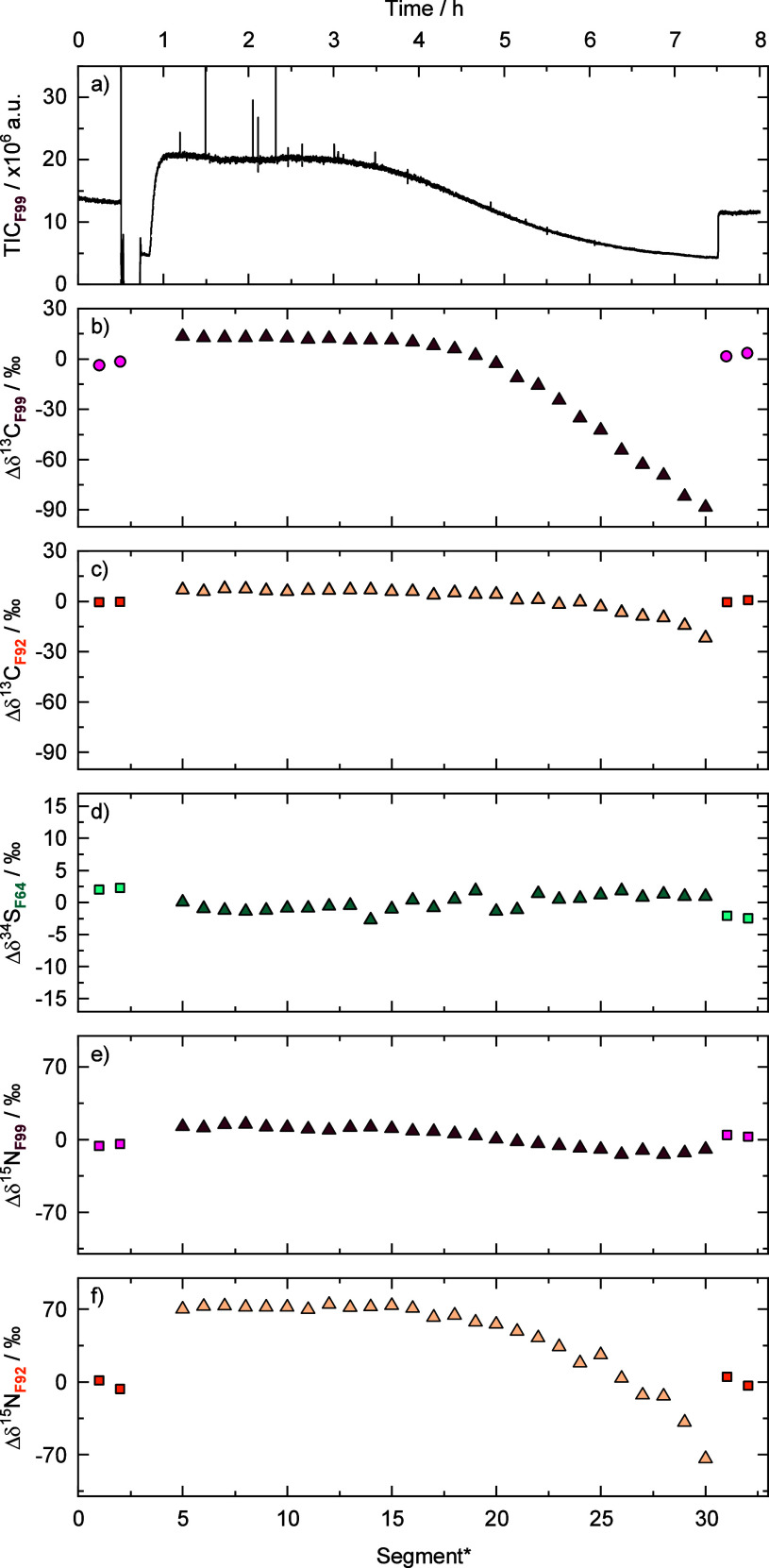
Homogenization of the captured SMX peak after LC. (a) Representative TIC over time for F99 when measuring the reference from the syringe (4 μM) at the beginning and the end, and a sample from the homogenized captured peak (injection of 40 μL of a 100 μM SMX solution) in between. Panels (b–f) show the resultant isotope values (Δδ^13^C, Δδ^34^S, and Δδ^15^N) when referenced against the average isotope value from the reference for each fragment; *each block was divided into segments of 15 min of data acquisition.

Our approach, therefore, delineates a successful way forward to eliminate effects from isotope fractionation during chromatography and concentration differences within the peak. First, the measurement window of the stable TIC directly after homogenization can be used for isotope measurements without the need for linearity correction. Second, thanks to homogenization, it is not necessary to integrate over the entire peak for 3 h to account for chromatographic artifacts; instead, analysis can be stopped once the necessary precision has been reached. Third, switching between a sample and a reference SMX solution from the syringe can be freely adapted to correct instrumental drifts, without the danger of losing valuable information while switching (Figure S5).

### Accuracy of the Hyphenation Setup

To assess the accuracy of our optimized mixing chamber approach, we used two standard solutions enriched with 13.2 ± 0.5‰ and 18.2 ± 0.5‰ at one carbon atom in F99, respectively. The calibration was performed by measuring the two position-enriched standards with different isotopic signatures (SMX_F99_1 and SMX_F99_2) and the one at natural abundance (SMX_F99_0) on five individual days. First, we used SMX_F99_0 as an anchor and reported SMX_F99_1 and SMX_F99_2 against these values (Figure 5a). Here, on measurement day three, both standard solutions showed a deviation from the expected value. However, a two-point calibration using SMX_F99_0 and SMX_F99_2 successfully compensated for this scale expansion (Figure 5c). For δ^13^C measurements in F99, we found 95% CIs of 1.5‰ using this setup.

**Figure 5:**
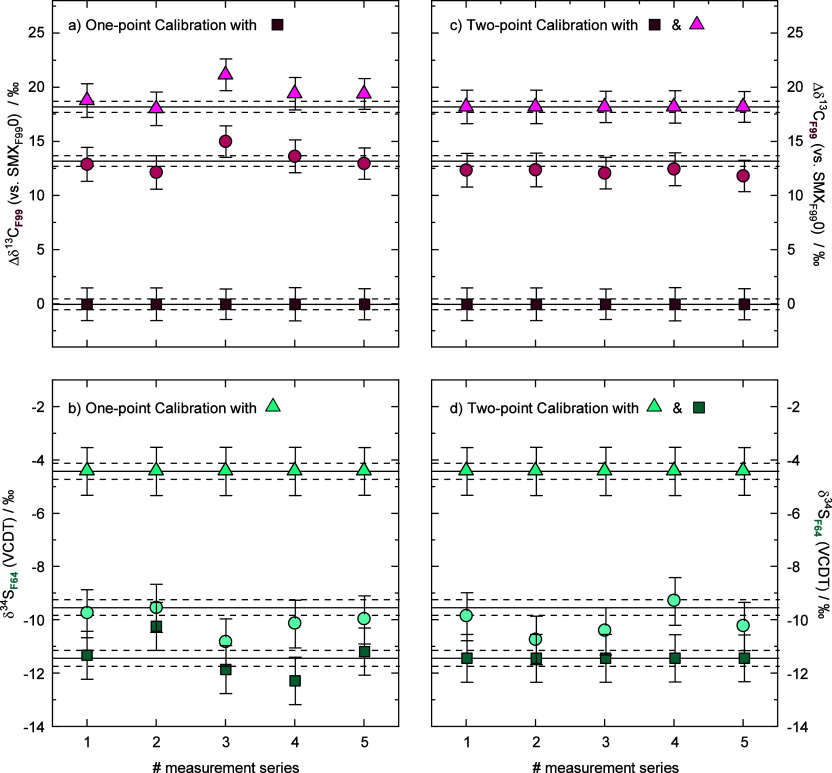
Accuracy of Δδ^13^C in F99, and of δ^34^S in F64, on five different days by comparing HPLC-ESI-Orbitrap-MS data to GC-IRMS and EA-IRMS data, respectively. (a + c) Δδ^13^C in F99 and δ^34^S in F64 using a one-point calibration, (b + d) Δδ^13^C in F99 and δ^34^S in F64 using a two-point calibration. The uncertainties represent the 95% confidence intervals of the quadruplicate drift-corrected values from one injection. The horizontal black lines indicate the isotope value determined by GC-IRMS or EA-IRMS with their respective uncertainties (black dashed lines) 0.5‰ for δ^13^C and 0.3‰ for δ^34^S.

Standardization of δ^34^S in F64 was performed using SMX from three different batches (SMX_F64_0, SMX_F64_1, and SMX_F64_2), which were characterized by EA-IRMS to span a calibration range of 7‰. The results using only one standard (−4.4‰) as an anchor show correct isotope values except on day 3 (Figure 5b). While in this case, the two-point calibration was not fully effective in reducing the scatter of all measurement days (Figure 5d), we note that the isotopic signatures between SMX_F64_1 and SMX_F64_2 differed by only 1.9‰. This is similar in magnitude to the 95% CIs of 0.9‰ we achieved for δ^34^S in F64, and is, hence, a result that can be expected within the given precision. Results obtained for δ^33^S are further consistent with isotope values that would be expected for mass-dependent fractionation (Table S2); however, again within a precision that makes it difficult to distinguish the different batches within our calibration range (Figure S7).

## Conclusion

ESI-Orbitrap-MS proved a powerful technique for measuring fragment- and position-specific isotope ratios of the antibiotic sulfamethoxazole as a polar organic model compound. To isolate SMX prior to isotope analysis, the present study brings forward homogenization of captured chromatographic peaks in a dynamic mixing chamber as an essential step in the hyphenation of HPLC with ESI-Orbitrap-MS. Applying this setup, we successfully measured δ^13^C and δ^34^S values in respective fragments of SMX with 95% CIs of 1.5‰ and 0.9‰, respectively, using only 4 nmol of target analyte for analysis of each fragment. In contrast, our findings demonstrate that the use of a capillary loop to capture the target analyte presents challenges, including concentration effects and isotope fractionation during chromatography, which limit the ability to correct for instrumental drifts. Here, hyphenation via our dynamic mixing chamber provided the solution to multiple problems. First, artifacts from linearity and chromatographic isotope effects were eliminated, enabling precise measurements. Second, this allowed for frequent switching between the sample and a reference solution to correct for instrumental drifts, because the need to closely follow isotope trends across a chromatographic peak was eliminated. Third, this greatly shortened measurement time, since analyses could be stopped once the necessary counting statistics had been reached. Fourth, this enables the measurement of different fragments from SMX during a single sample injection, as the remaining time can be used to run analyses in different ionization modes and mass selections. The new approach, therefore, paves the way for the development of similar methods in HPLC-Orbitrap-MS for other compounds, advancing future research across various fields and promising to enable more informative isotopic studies.

## Supplementary Material



## Data Availability

The raw data supporting the findings of this study are available at 10.5281/zenodo.17061171.

## Notes

Standard preparation, GC-IRMS analysis, EA-IRMS analysis, fragmentation pattern of sulfamethoxazole, automation of peak capturing, analysis sequence of a linearity experiment, comparison of injection methods, data processing, error propagation, shot noise limit, Allan deviation, and δ^33^S values (PDF)
