# Supplementary material for: Coupling Liquid Chromatography to Orbitrap Isotope Ratio Mass Spectrometry: Overcoming Isotope Effects of Chromatography and Amount-Dependency by Peak Homogenization
Source: Anal Chem. 2025 Dec 19;98(1):590–600. doi: 10.1021/acs.analchem.5c05530 (PMC12809655; doi:10.1021/acs.analchem.5c05530)
Supplement: Supplementary file 1 [file ac5c05530_si_001.pdf]

Coupling Liquid Chromatography to Orbitrap Isotope Ratio Mass Spectrometry: Overcoming Isotope Effects of Chromatography and Amount-Dependency by Peak Homogenization

Aoife Canavan[a], Leonhard Prechtl[a], Habib Al-Ghoul[a], Nils Kuhlbusch[b,c], Andrea M. Erhardt[d], Martin Elsner[a,*]

[a]Technical University of Munich, TUM School of Natural Sciences, Department of Chemistry, Chair of Analytical Chemistry and Water Chemistry, Lichtenbergstraße 4, 85748 Garching, Germany

[b]Thermo Fisher Scientific (Bremen) GmbH, Hanna-Kunath-Straße 11, 28199 Bremen, Germany

[c]University of Münster, Department of Inorganic and Analytical Chemistry, Corrensstraße 48, 48149 Münster, Germany

[d]University of Kentucky, Department of Earth and Environmental Sciences, 121 Washington Ave, Lexington, Kentucky 40506, USA

*Corresponding author: Martin Elsner
Address: Lichtenbergstraße 4, 85748 Garching b. München
Phone: +49 (89) 289 - 54500
E-mail: m.elsner@tum.de

Contents

S1 Standard Preparation S-2
S2 GC-IRMS for $\delta^{13}C$ Analysis of SMX S-2
S3 EA-IRMS for $\delta^{34}S$ Analysis of SMX S-3
S4 Fragmentation Pattern of Sulfamethoxazole S-4
S5 Automation of Peak Capturing by a Trigger of the DAD S-5
S6 Analysis Sequence of a Linearity Experiment S-6
S7 Comparison of Injection Methods S-6
S8 Details on Data Evaluation S-6
S9 Data Processing using the Setup with the Dynamic Mixing Chamber S-8
S10 Error Propagation using the Setup with the Dynamic Mixing Chamber S-8
S11 Shot Noise Limit and Allan Deviation for Analyzed Fragments S-10
S12 $\delta^{33}S$ Values in F64 .... S-11
S13 References S-11

S1 Standard Preparation

For carbon isotope analysis, SMX with a ^{13}C label at one position within the isoxazole moiety was used.[1] The synthesis product was purified by HPLC. These HPLC measurements were carried out on a Nexera XR HPLC system equipped with two solvent pumps (LC-20AD), an autosampler (SIL-20AC XR), a column oven (CTO-20A prominence), a diode array detector (SPD-M20A prominence), and a fraction collector (FRC-10A) (all, Shimadzu, Japan). An Xterra RP18 column (particle size = 3.5 µm, L × D = 150 × 3.0 mm, pore size = 125 Å, Waters, Germany) was used for chromatographic separation with the column oven temperature set to 40 °C and using a flow rate of 0.5 mL min^{-1}. As the mobile phase, a binary gradient consisting of water (A) and methanol (B) was used. The gradient started with 10% B for 1 min, then increased to 90% B within 9 min, where it was held at 90% B for 1 min.

To this end, 45 µL of a ^{13}C-labeled SMX solution in methanol/water (~ 25 mg L^{-1}) was injected, and the fraction containing the ^{13}C-labeled SMX was collected. Subsequently, the solvent of the collected fraction was evaporated under a gentle stream of nitrogen (1 mL min^{-1}, 50 °C, TurboVapLV, Biotage, Sweden), and the residue was reconstituted in 910 µL of methanol. To create the in-house standard SMXF$_{99}$1, 300 µL of the reconstituted solution was added to a vial containing 500 µL of a 1 g L^{-1} solution of SMX (Lot: BCCH3594) at natural isotopic abundance in methanol. This corresponded to a proportion labeled to non-labeled SMX of approximately 1:135. Additional methanol was added to reach the target volume of 1 mL. To create the in-house standard SMXF$_{99}$2, the same procedure was applied, only with the addition of 450 µL of the reconstituted solution corresponding to a proportion of 1:900.

S2 GC-IRMS for δ^{13}C Analysis of SMX

The δ^{13}C isotope ratio measurements of SMX were conducted on a GC-IRMS system consisting of a TRACE 1310 gas chromatograph hyphenated to a Finnigan MAT 253 isotope ratio mass spectrometer via an IsoLink II and a ConFlo IV interface (all, Thermo Fisher Scientific, Germany). For the analysis, samples of 1.5 µL were injected by a TriPlusRSH (Thermo Fisher Scientific, Germany) into a split/splitless injector with a split ratio of 1:4 equipped with a liner (4 mm ID × 78.5 mm length, 800 µL, ultra inert, double taper, without glass wool, Agilent Technologies, USA) maintained at a temperature of 280 °C. The target analytes were separated at a constant helium flow rate of 2 mL min^{-1} on an Agilent J&W DB-1 column (30 m length × 0.32 mm ID × 1.0 µm film thickness, Agilent Technologies, USA), which was enclosed by a pre-and post-column (each 1 m deactivated fused silica guard column, 0.32 mm ID, Agilent, USA). The GC oven program started at 170 °C, followed by a ramp to 320 °C with a rate of 20

°C min^{-1}, where the final temperature was held for 5 min. Upon elution from the column, the target analytes underwent combustion to CO_2 and subsequent reduction of nitrogen oxides to N_2 at a temperature of 1000 °C using commercial combustion reactors (NiO tube CuO − NiO reactor, 2 mm, Thermo Fisher Scientific, Germany). The oxidation capacity of the reactor was ensured before each measurement by a seed oxidation for 15 s, followed by a subsequent backflush period of 2 min to remove any undesired oxidation products. To ensure system stability and method accuracy, retention times and isotope values were constantly monitored, and samples were bracketed with international reference material (caffeine, USGS62). The limit of precise isotope analysis and the method's trueness were determined using varying concentrations of SMX (2 nmol C to 20 nmol C on column). The software Isodat (Thermo Fisher Scientific, Germany) automatically performed peak detection and integration using the individual background algorithm for baseline correction. The method quantification limits were determined with the moving mean procedure according to Jochmann et al.[2]. The results for the three prepared SMX solutions ($SMX_{F99}0$, $SMX_{F99}1$, and $SMX_{F99}2$) are summarized in Table S1. Assuming that the enrichment in ^{13}C only occurs in F99, the enrichment in F99, $\Delta\delta^{13}C_{SMX\_F99}$, was calculated by equation (S1), where $\delta^{13}C_{enriched}$ is either $\delta^{13}C_{SMX\_F99\_1}$ or $\delta^{13}C_{SMX\_F99\_2}$.

$$\delta^{13}C_{SMX_{F99}} = (\delta^{13}C_{enriched} - \delta^{13}C_{SMX_{F99\_0}}) \cdot \frac{10}{4} \tag{S1}$$

Table S1: Summary of $\delta^{13}C$ values by GC-IRMS.

Chemical	Abbreviation	$\delta^{13}C$ / ‰	$\Delta\delta^{13}C_{F99}$ / ‰ [a]
Sulfamethoxazole	$SMX_{F99}0$	-28.4 ± 0.3	0.0 ± 0.5
Sulfamethoxazole	$SMX_{F99}1$	-23.1 ± 0.3	13.2 ± 0.5
Sulfamethoxazole	$SMX_{F99}2$	-21.1 ± 0.3	18.2 ± 0.5

[a] calculated according to Equation S1

S3 EA-IRMS for $\delta^{34}S$ Analysis of SMX

For the determination of the $\delta^{34}S$ in SMX from different batches, SMX was weighed into tin capsules, and tungsten oxide was added as a catalyst. The samples were analyzed by EA-CF-IRMS. The samples were introduced into a glass column containing tungsten oxide as an oxidizing agent and electrolytic copper as a reducing agent, maintained at a temperature of 1020 °C. After separation of the product SO_2 gas through GC, the respective $\delta^{34}S$ values were measured by IRMS. International Reference materials, such as barium sulfate and silver sulfide, were used for calibration. Assuming mass-dependent fractionation, the following $\delta^{33}S$ values were estimated according to equation S2[3]:

$$\delta^{33}S_{SMX_{F64}} = 0.515 \cdot \delta^{34}S_{SMX_{F64}} \tag{S2}$$

Table S2: Summary of $\delta^{34}S$ values by EA-IRMS and calculated $\delta^{33}S$ values by equation S2.

Chemical	Abbreviation	Lot number	$\delta^{34}S$ / ‰	$\delta^{33}S_{calc}$ / ‰ [a]
Sulfamethoxazole	$SMX_{F64}0$	BCBB6435	-4.4 ± 0.3	-2.3 ± 0.3
Sulfamethoxazole	$SMX_{F64}1$	BCCH3594	-9.5 ± 0.3	-4.9 ± 0.3
Sulfamethoxazole	$SMX_{F64}2$	BCCJ8018	-11.4 ± 0.3	-5.9 ± 0.3

[a] calculated according to Equation S2

S4 Fragmentation Pattern of Sulfamethoxazole

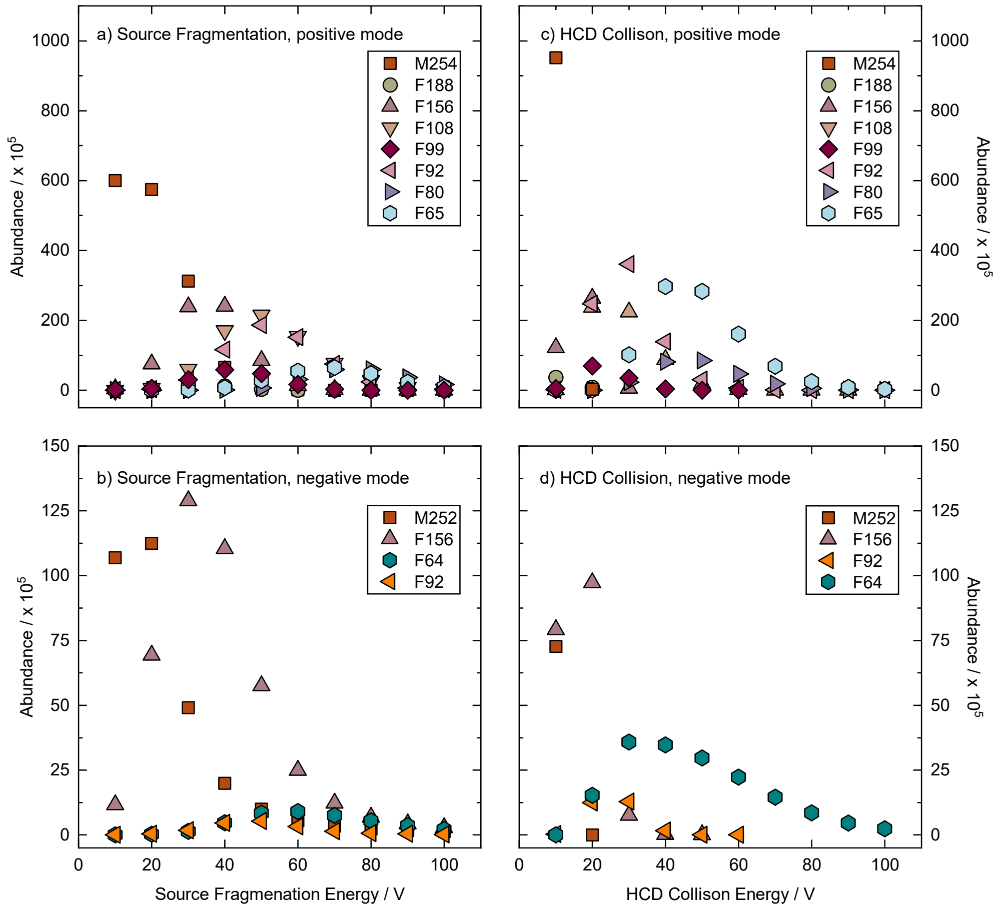

Figure S1: Abundances of SMX and SMX fragment ions at different source fragmentation and HCD collision energies after positive or negative ionization by directly infusing an SMX solution (5 µM, 36% MeOH in H_2O with 0.1% formic acid); the molecular ion is abbreviated with M and the fragment ions with F, followed by the resultant m/z value.

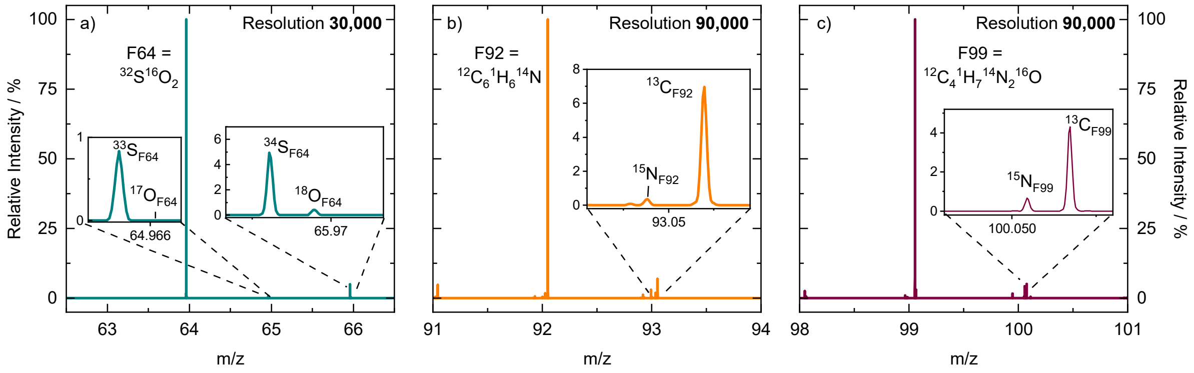

Figure S2: Representative mass spectra for a) F64, b) F92, and c) F99 with the instrument parameters used; mass regions corresponding to the rare isotopologues are magnified; the peaks of the rare isotopologue satellites are represented by their respective fragment indices, for example, $^{34}S_{F64}$ corresponds to the peak of $^{34}S^{16}O_2$.

S5 Automation of Peak Capturing by a Trigger of the DAD

First, the retention time of the target analyte, SMX, was determined. Approximately 0.5 minutes before the start of the peak, a trigger was set to monitor the UV signal at a wavelength of 258 nm. When the UV signal increased by more than 10 units with a true time of 5, the trigger activated a change in the position of Valve 1. The valve then switched back after 80 seconds, which ensured complete capture of the peak. Subsequently, the relay of the nano pump (note: any relay from any module in this setup can be used) was activated for 60 seconds, which activated stirring inside the dynamic mixing chamber. After the homogenization, the nano pump was started at a flow rate of 4 mL min^{-1}. It is essential to maintain a sufficient volume between the DAD and Valve 1 to prevent loss of the peak's beginning. Therefore, we used a peek capillary (ID 0.250 mm, length 4 m). This connecting capillary should generate as little back pressure as possible (see the specifications of the installed DAD flow cell) while avoiding any peak broadening.

S6 Analysis Sequence of a Linearity Experiment

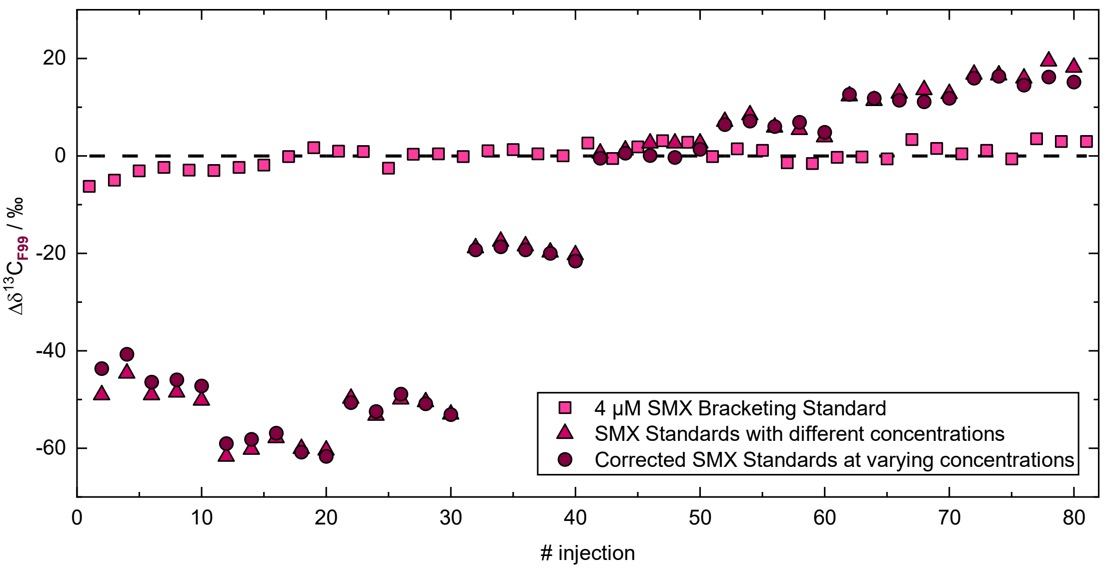

Figure S3: Analysis sequence of F99 of an SMX standard, altering SMX at 4 µM, and eight SMX solutions at different concentrations to investigate system drift.

S7 Comparison of Injection Methods

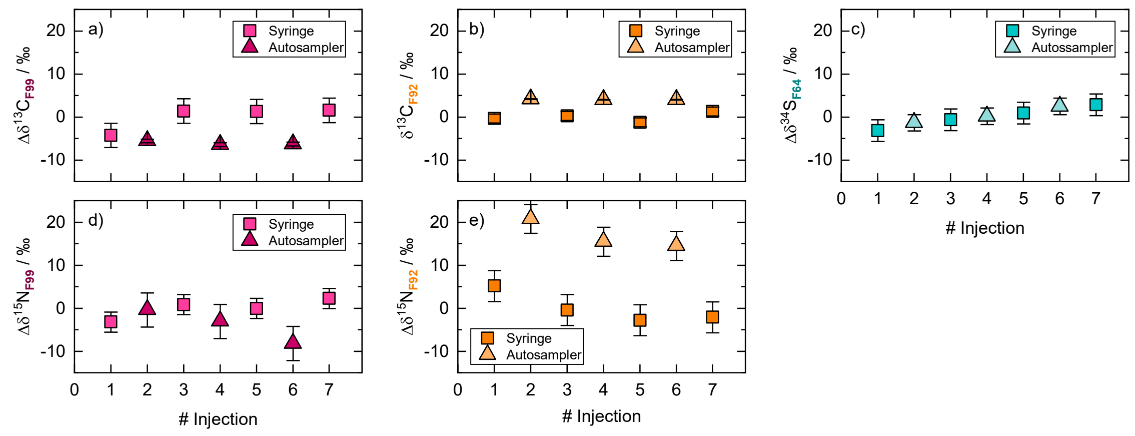

Figure S4: Comparison of two injection methods by injecting the same SMX solution (5 µM) via the syringe and the autosampler in the absence of a chromatographic column, showing isotope data of carbon (a) and nitrogen (d) in F99, carbon (b) and nitrogen (e) in F92, and sulfur (c) in F64; the error bars represent the standard deviation of the triplicate or quadruplicate measurement of autosampler or syringe injection, respectively.

S8 Details on Data Evaluation

For the *linearity experiment* (Figure 2) based on autosampler injections, the average isotope ratio of the 4 µM SMX solution applied before and after each concentration measurement was used as the $R_{reference}$ (an example is provided in Figure S3), with its $\delta^{h}E_{\text{reference}}$ value set to 0‰, expressing isotope ratios as deviations from the 4 µM SMX solution. In these experiments, the

uncertainties represent estimates of the standard deviation derived from the quintuplicate measurement of each concentration.

For the *comparison of the injection techniques* (Figure S4), the average of the four 5 µM syringe injections was used as $R_{reference}$, which was set to $\delta^{h}E_{reference}$ = 0‰. Both the individual syringe injection and autosampler injection values were expressed as deviations from the 5 µM SMX syringe average. Here, the error bars represent estimates of the standard deviation derived from the triplicate of the autosampler injection and the quadruplicate of the syringe injections.

For *peak capturing experiments* (Figure 3, 4) using a capillary loop and dynamic mixing chamber, respectively, the average of all segments from the four syringe injections was used as the $R_{reference}$, with its $\delta^{h}E_{reference}$ set to 0‰. We note that in the experiment of Figure 4, no bracketing was performed in order not to lose any information about the trend over time. The reported uncertainties indicated in the segment data are estimates derived by lumping comparable data points, for example, the standard deviation over all data points acquired from the reference and of each data point from the triplicate sample injection.

In the *validation of the final setup using the dynamic mixing chamber* (Figure 5), where measurements were conducted of SMX standards with different isotope values, each homogenized standard was bracketed with SMX from the syringe. Here, we used a switching time of 5 minutes, meaning that the first 5 minutes of each block were not used for data evaluation (Figure S5). The $\delta^{13}C$ value of $SMX_{F99}0$ was set to 0, and the other standards are reported relative to this value. Sulfur values, on the other hand, are reported on the international scale since only one sulfur atom is present in the molecule. The 95% CI was determined for carbon and sulfur isotope ratios, respectively (Section S10).

S9 Data Processing using the Setup with the Dynamic Mixing Chamber

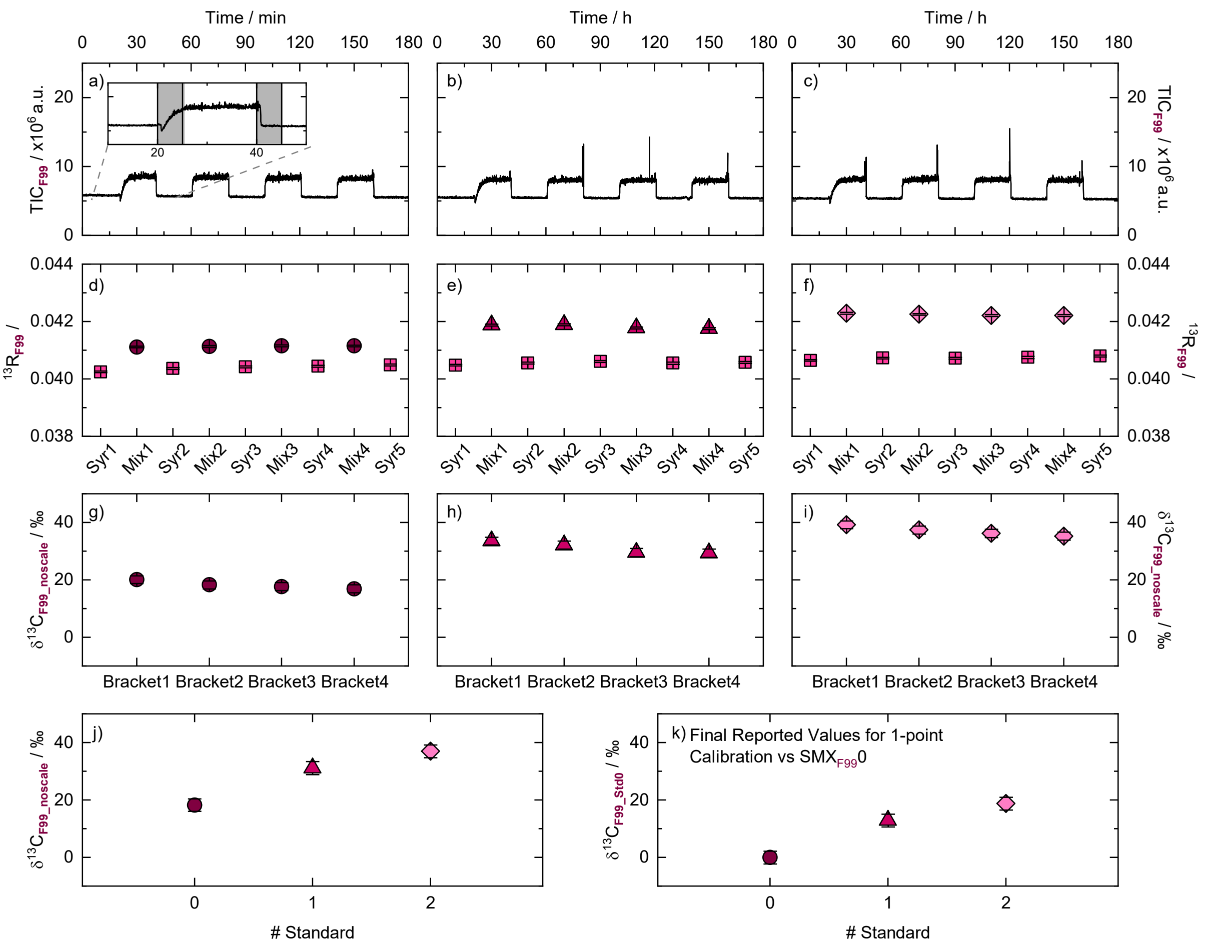

Figure S5: Data evaluation using the setup with the dynamic mixing chamber. TIC of F99 over the 3-hour measurement time for the analysis of $SMX_{F99}0$ (a), $SMX_{F99}1$ (b), and $SMX_{F99}2$ (c), bracketed with a syringe containing 4 µM $SMX_{F99}0$. In Figure a) a zoom-in is shown, highlighting the 5-minute blocks in grey from which data was discarded (switching time). Panels d) to f) show $^{13}R_{F99}$ for the respective 15-minute blocks for $SMX_{F99}0$ (d), $SMX_{F99}1$ (e), and $SMX_{F99}2$ (f), where the uncertainties represent the standard error of the mean. The drift-corrected $\delta^{13}C$ values obtained using syringe measurements before and after are given for $SMX_{F99}0$ (g), $SMX_{F99}1$ (h), and $SMX_{F99}2$ (i); error bars represent propagated errors. j) $\delta^{13}C$ values of the average of the quadruplicate measurements of panels g) to i), where uncertainties represent 95% confidence intervals, k) Final reported $\delta^{13}C$ values against $SMX_{F99}0$, where uncertainties represent 95% confidence intervals.

S10 Error Propagation using the Setup with the Dynamic Mixing Chamber

For the calculation of the $R_{reference}$, the average of the ratio from the syringe infusion measured before (Std1) and after (Std2) the sample from the dynamic mixing chamber was calculated. The error of this averaged ratio was estimated using equation S3, which accounted for the standard error of the mean of each respective ratio.

$$\sigma_{reference-average} = \sqrt{\left(\frac{\sigma_{Std1}}{2}\right)^2 + \left(\frac{\sigma_{Std2}}{2}\right)^2} \quad \text{(S3)}$$

The standard error of the mean of R_{sample}, σ_{sample}, and $\sigma_{reference}$ were propagated using the Gaussian error propagation (eq. S4) and are stated in ‰.

$$\sigma_{\delta-value} = \sqrt{\left(\frac{1000 \cdot \sigma_{sample}}{R_{reference-average}}\right)^2 + \left(\frac{1000 \cdot R_{sample} \cdot \sigma_{reference-average}}{R_{reference-average}^2}\right)^2} \quad \text{(S4)}$$

Such errors were obtained for each reference sample bracketing and further propagated by equation (S5), resulting in 95% CI:

$$\sigma_{final\delta-value-95\%CI} = \left(\frac{1}{n} \cdot \sqrt{\sum_{i=1}^{n} \sigma_i^2}\right) \cdot t_{\alpha,n-1} \quad \text{(S4)}$$

Here, $t_{\alpha,n-1}$ is the Student t-factor, which resulted in a Student t-factor of 3.182 in our case for four samples and five reference blocks.

S11 Shot Noise Limit and Allan Deviation for Analyzed Fragments

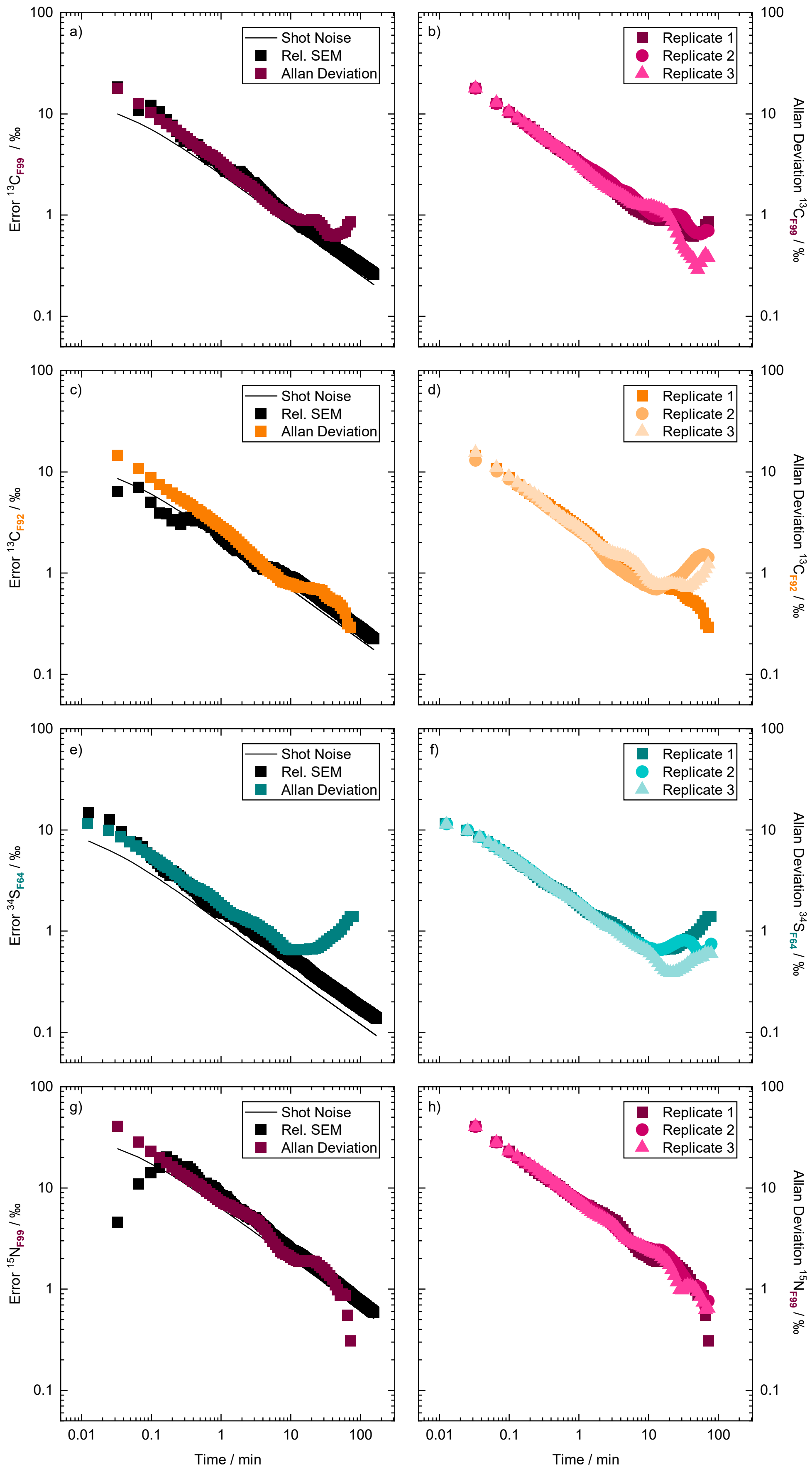

Figure S6 (continued on the next page)

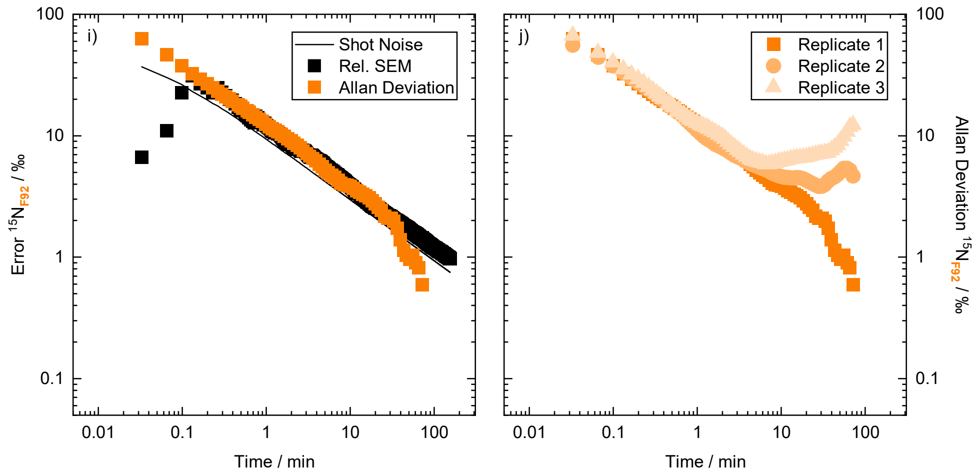

Figure S6: Left panels: representative estimated shot noise, relative standard error of the mean, and Allan Deviation from the respective second syringe infusion (main manuscript Figure 3). Right panels: Allan Deviation from three replicates (second, third, and fourth reference measurement, main manuscript Figure 3), specifically: a+b) for ^{13}C in F99, c+d) for ^{13}C in F92, e+f) for ^{34}S in F64, g+h) for ^{15}N in F99, and i+j) for ^{15}N in F92.

S12 $\delta^{33}S$ Values in F64

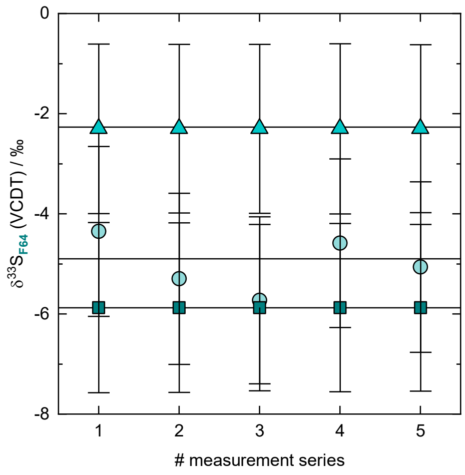

Figure S7: $\delta^{33}S$ values in F64 on five different days by HPLC-ESI-Orbitrap-MS using a two-point calibration. The error bars represent 95% CIs. The horizontal black lines indicate the isotope value calculated from the EA-IRMS values, assuming mass-dependent fractionation.

S13 References

(1) Canavan, A.; Dirr, C.; Elsner, M. Intramolecularly Labeled Reference Standards of Sulfamethoxazole for Fragment-Specific Isotope Analysis by Electrospray Ionization Orbitrap Mass Spectrometry. - *submitted*. 2025.
(2) Jochmann, M. A.; Blessing, M.; Haderlein, S. B.; Schmidt, T. C. A new approach to determine method detection limits for compound-specific isotope analysis of volatile organic compounds. *Rapid Commun. Mass Spectrom.* **2006**, *20* (24), 3639-3648.
(3) Ono, S.; Wing, B.; Rumble, D.; Farquhar, J. High precision analysis of all four stable isotopes of sulfur (^{32}S, ^{33}S, ^{34}S and ^{36}S) at nanomole levels using a laser fluorination isotope-ratio-monitoring gas chromatography-mass spectrometry. *Chem. Geol.* **2006**, *225* (1-2), 30-39.
